# The miR-133a, TPM4 and TAp63γ Role in Myocyte Differentiation Microfilament Remodelling and Colon Cancer Progression

**DOI:** 10.3390/ijms22189818

**Published:** 2021-09-10

**Authors:** Sabrina Caporali, Cosimo Calabrese, Marilena Minieri, Massimo Pieri, Umberto Tarantino, Mario Marini, Stefano D’Ottavio, Silvia Angeletti, Alessandro Mauriello, Claudio Cortese, Sergio Bernardini, Alessandro Terrinoni

**Affiliations:** 1 Department of Industrial Engineering, University of Rome Tor Vergata, 00133 Rome, Italy; sabrina.caporali93@gmail.com; 2 Department of Experimental Medicine, University of Rome Tor Vergata, Via Montpellier 1, 00133 Rome, Italy; doccosimo@libero.it (C.C.); minieri@med.uniroma2.it (M.M.); massimo.pieri@uniroma2.it (M.P.); alessandro.mauriello@uniroma2.it (A.M.); claudio.cortese@uniroma2.it (C.C.); bernardini@med.uniroma2.it (S.B.); 3 Department of Clinical Sciences and Translational Medicine, University of Rome Tor Vergata, Via Montpellier 1, 00133 Rome, Italy; umberto.tarantino@uniroma2.it (U.T.); stefano.dottavio@uniroma2.it (S.D.); 4 Centre of Space Biomedicine and Department of Systems Medicine of the University of Rome Tor Vergata, 00133 Rome, Italy; mario.marini@uniroma2.it; 5 Unit of Clinical Laboratory Science, University Campus Bio-Medico of Rome, Via Alvaro del Portillo, 00128 Rome, Italy; s.angeletti@unicampus.it

**Keywords:** miRNAs, physical activity, colon carcinoma (CRC), TAp63, Tropomyosins, miR-133a, TPM4, circulating miRs

## Abstract

MicroRNAs (miRNAs) play an essential role in the regulation of a number of physiological functions. miR-133a and other muscular miRs (myomiRs) play a key role in muscle cell growth and in some type of cancers. Here, we show that miR133a is upregulated in individuals that undertake physical exercise. We used a skeletal muscle differentiation model to dissect miR-133a’s role and to identify new targets, identifying Tropomyosin-4 (TPM4). This protein is expressed during muscle differentiation, but importantly it is an essential component of microfilament cytoskeleton and stress fibres formation. The microfilament scaffold remodelling is an essential step in cell transformation and tumour progression. Using the muscle system, we obtained valuable information about the microfilament proteins, and the knowledge on these molecular players can be transferred to the cytoskeleton rearrangement observed in cancer cells. Further investigations showed a role of TPM4 in cancer physiology, specifically, we found that miR-133a downregulation leads to TPM4 upregulation in colon carcinoma (CRC), and this correlates with a lower patient survival. At molecular level, we demonstrated in myocyte differentiation that TPM4 is positively regulated by the TA isoform of the p63 transcription factor. In muscles, miR-133a generates a myogenic stimulus, reducing the differentiation by downregulating TPM4. In this system, miR-133a counteracts the differentiative TAp63 activity. Interestingly, in CRC cell lines and in patient biopsies, miR-133a is able to regulate TPM4 activity, while TAp63 is not active. The downregulation of the miR leads to TPM4 overexpression, this modifies the architecture of the cell cytoskeleton contributing to increase the invasiveness of the tumour and associating with a poor prognosis. These results add data to the interesting question about the link between physical activity, muscle physiology and protection against colorectal cancer. The two phenomena have in common the cytoskeleton remodelling, due to the TPM4 activity, that is involved in stress fibres formation.

## 1. Introduction

Skeletal muscles represent the largest tissue mass of the body and can be distinguished from smooth muscles as they are composed of striated myofibers of multinucleated syncytia, coming from the fusion of myocytes. Skeletal muscle fibres are surrounded by a thin basal lamina composed of glycoproteins such as laminin and collagen fibrils, which ensure stability and support to the muscle fibres. An impaired muscle function is common to a number of clinical conditions, like developmental disorders, rhabdomyosarcoma, or muscular dystrophies [1]. In adults, aging or sarcopenia could give rise to important muscular impairment, thus deteriorating of the quality of life. Therefore, a better understanding of the myogenetic process could clarify the mechanisms of muscle disease and regeneration. On the other hand, skeletal muscle function relies on the cell’s cytoskeleton and cytoskeletal proteins are also responsible for proper functioning of cellular and biochemical processes, by regulating molecular trafficking, as well as cell structure and motility. In physiological conditions, the cytoskeletal network confers to cells resistance to deformation [2]. In malignant cells, a reorganisation of the cytoskeleton has been detected [3]. In fact, reprogramming of the cytoskeletal network composition and structure during carcinogenesis is necessary to support cancer progression by promoting tumour cell survival, growth, and moreover to permit the invasion of other tissues [4]. The modifications occurring during transformation involves different cytoskeletal types and their associated molecules, within these, the microfilaments and actin stress fibres [5]. The microfilaments are mainly made up of actin [6], that can exist as a globular monomer, or as a filamentous polymer. Actin polymerisation and depolymerisation are finely tuned to maintain cell morphology, adhesion, and motility. Indeed, the modification of the actin cytoskeleton during tumorigenesis is able to promote tumour formation, survival, and metastasis [6]. Actin filaments are cross-linked by α-actinin, interacting with myosin to form actomyosin bundles, the actin stress fibres. These are fundamental for cell adhesion to the extracellular matrix (ECM), and in tumour cells a depletion of these fibres stimulate cell migration [7]. Importantly actin filaments can be also associated to different tropomyosin (TPM) proteins. This family comprises four different muscular proteins, TPM1, TPM2, TPM3 and TPM4. All of them can bind actin filaments in both muscle and non-muscle cells, allowing muscle contraction and cytoskeleton stability, respectively. Indeed, actin-TPM4 microfilaments are important for contraction, cell morphology, cell motility, and intracellular vesicle transport. TPM4 is able to promote cell migration modulating F-actin assembly in lung cancer [8], moreover, high levels of TPM4 have been detected in HCC patients with distant metastasis. The overexpression of this tropomyosin seems to enhance not only cell migration but also viability by negative SUSD2 regulation [9].

The study of skeletal muscle development and differentiation could give valuable information about the molecular players involved in the regulation of the different microfilament proteins, and the knowledge transferred to the cytoskeleton rearrangement observed in cancer cells. Muscle development and remodelling depend upon microRNA regulation [10,11], which is also important for cancer development and progression [12]. A number of publications report microRNAs miR-1, miR-133a, and miR-206 as muscle specific. In particular, miR-1 and miR-206 promote myoblast differentiation into myotubes, as confirmed by the increased expression of myogenin and myosin heavy chain (MHC), respectively, early and late myogenic markers [13], while miR-133a is involved in myoblast proliferation [13,14,15]. In vitro, human skeletal myoblasts transfected with miR-133a inhibitor showed decreased proliferation and increased differentiation, as confirmed by the increased expression of sarcomeric α-actinin protein [14]. These data suggest that miR-133a inhibition could induce myotube formation. The overexpression of miR-133a represses myogenin and MHC expression, maintaining myoblasts in a proliferating state [13]. In contrast, C2C12 myoblasts transfected with miR-1 and miR-206 show accelerated differentiation [14]. In vivo, miR-133a knockout mice show mitochondrial dysfunction, centronuclear myopathy and disorganisation of T-tubules in muscle fibres [16]. In cancer, miRs can have a dual function acting as onco-miR or tumour suppressor [17], and the alteration of miRNA expression has been associated with initiation and development of lung, breast, colon and other cancer types [18].

Moreover, some c-myomiRs (circulating myomiRs) have been correlated with different stages of cancer development and therapeutic response [19]. In different type of cancers, the levels of circulating myomiRs have been associated to an unfavourable prognosis and survival [20]. Indeed, the downregulation of miR-133 levels in serum has been found in patients with breast cancer, related to disease progression and poor prognosis [21]. Among this wide group, miR-133a, miR-133b, and miR-206 showed possible implications in tumorigenesis and tumour progression of osteosarcoma, and they have been found frequently downregulated in various types of cancers [22,23]. Deng et al. reported a downregulation of miR-206 in gastric cancer and an upregulation of its direct target MUC1, responsible for invasion and metastasis through the activation of the WNT signalling pathway. MiR-206 re-expression suppressed proliferation, migration, metastasis, and increased apoptosis. Moreover, high miR-206 expression levels have been correlated to better prognosis [24].

Moreover, downregulation of miR-133 has been reported in bladder cancer [24], oesophageal squamous cell carcinoma [25], hepatocellular carcinoma, lung carcinoma [26,27,28], and prostate cancer [29]. In pancreatic cancer, miR-133 low expression correlated with aggressive behaviour of tumour and poor survival [30]. In colon cancer (CRC), miR-133b seems to be downregulated [31,32], and it has been demonstrated that its upregulation inhibits RhoA and E-cadherin, blocking cell migration and invasion [33]. Notably, it has been demonstrated that this miR is driven by TAp63, the long isoform of the p63 protein. The downregulation of the latter has been correlated with metastatic phenotype in CRC [34].

Physical exercise is a potent activator of gene expression [35] and the information about modifications of miRNA transcription levels and the consequent regulation of their targets during training, could be useful not only to understand myocyte proliferation/differentiation processes, but could also provide fundamental knowledge on the players involved in microfilament remodelling, and its importance in cancer physiology. During physical exercise, a variation in the concentration of different c-miRNAs was demonstrated in both plasma and serum [36,37,38,39,40], and therefore, they may be detected as biomarkers.

Moreover, literature specific data show the ability of physical exercise to be protective against colon cancer (CRC) onset. Many data support a relationship between the increased levels of physical exercise with a decreased colon cancer risk by the regulation of the immune system regulating the secretion of hormones, prostaglandins and cholesterol concentration [41]. Physical exercise can also determinate serological changes, mainly in TNF-α, IL-6 and IL-8 cytokines associated with the reduction of colon cancer cell growth [42]. It would be interesting, in this study, to investigate a possible biological mechanism connecting physical exercise with the regulation of the myomiRs, and consequently of its target.

Here we investigated myo-miRs regulation during physical training, and analysed their role in myocyte development and differentiation, looking for new targets that could be relevant also for cancer physiology. We identified TPM4 as a new target of miR-133a and demonstrate its involvement in the regulation of myocyte differentiation and CRC progression. TPM4 is an integral part of actin cytoskeleton, particularly it is involved in stress fibre formation and reorganisation. Since the modification of cell cytoskeleton is a fundamental step for tumour invasiveness, it would be interesting to dissect the role of TPM4 also in CRC physiology. Importantly, this regulation also involves in myocytes the p63 transcription factor, specifically the TAp63 isoform.

## 2. Results

### 2.1. Blood Parameters Related to Physical Exercise and Analysis of C-MyomiRs in Plasma

A group of human subjects were trained as described in a previously published protocol [43]. After the first cycle of training, several blood parameters were measured to detect their modifications during training and to verify the principal markers correlated to muscular activity (see Supplementary Figure S2).

**Figure 1:**
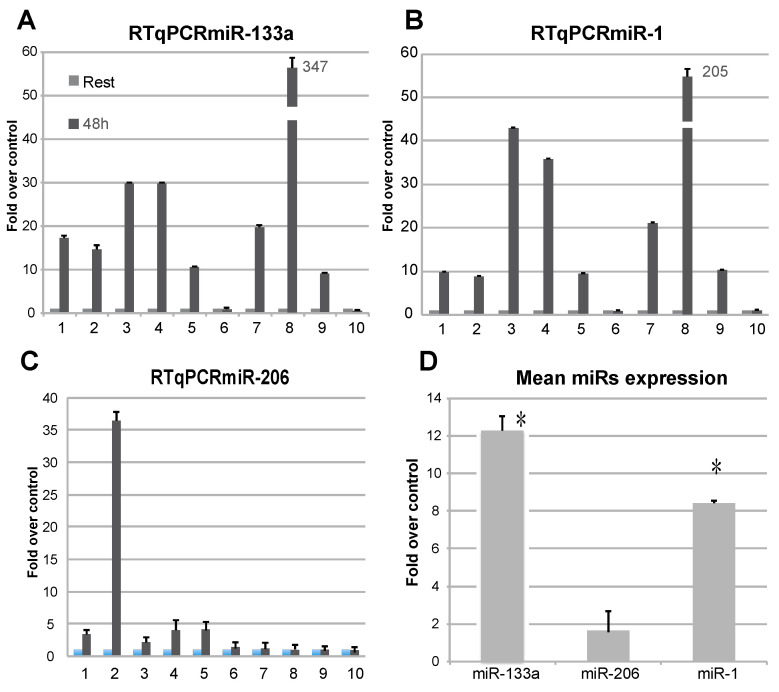
Analysis of the presence of c-myomiRs from RNA extracted from plasma subjects. Levels of miR-133a (**A**), miR-1 (**B**), and miR-206 (**C**) were analysed by RTqPCR and presented as fold-over-control. Upregulation of miR-133a is detectable in most of the probands. The miR-206 seems not be widely regulated in this context. A mean value of expression was calculated for the presented miRs (**D**), showing miR-133a as the most upregulated. (*n* = 3, * *p* < 0.01 *t*-test, two tails, equal variance).

The analysis of c-myomiRs abundance in RNA isolated from plasma [44] of individuals subjected to electrostimulation exercise, as compared to T0 plasma as control, showed a differential expression of miR-1 and miR-133a (Figure 1A–D). As it is shown in Figure 1A, miR-133a showed an upregulation in nearly all the probands analysed, even if with different intensity, whereas miR-1 showed a less homogeneous upregulation (Figure 1B). On the other hand, miR-206 was regulated only in one sample (Figure 1C). In the analysis of miRs levels at 48 h as compared to resting conditions, miR-133a proved to be the most upregulated, suggesting that it is significantly regulated during physical exercise (Figure 1D).

### 2.2. TPM4 as Possible miR-133a Target

**Figure 2:**
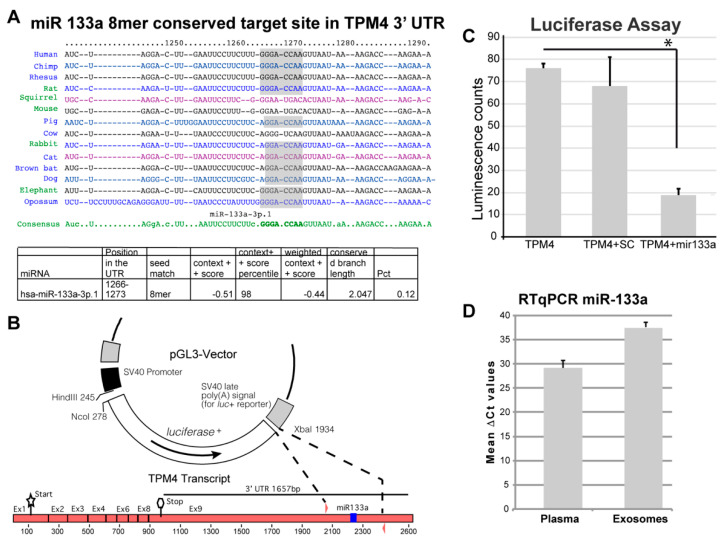
miR-133a putative target analysis. (**A**) The putative targets of miR-133a were analysed by means of the TargetScan-7.1 software and here are represented the consensus sequence and the conservation across different species [45]. A conserved putative binding site was retrieved in the 3′ UTR of the TPM4 transcript. (**B**) A 300 bp fragment, containing the putative responsive element, was cloned into the pGL3 vector, replacing part of the 3′ UTR of the luciferase gene, this will permit to control the luciferase gene if the cloned sequence contains a genuine binding site for miR-133a. (**C**) Assay showing the inhibition of luciferase when the vector is co-transfected with miR-133a demonstrates its interaction with the cloned responsive element (*n* = 6, * *p* < 0.01, *t*-test two tails, coupled). (**D**) miR-133a amplification from RNA extracted from plasma and from isolated exosomes. (*n* = 3).

The bioinformatic analysis (TargetScan) retrieved the gene coding for tropomyosin 4 (TMP4) as a novel putative target for miR-133a (Figure 2A). In fact, the 3′ UTR of the TMP4 transcript shows the presence of a putative binding site for miR-133a, which is conserved in different species (Figure 2A).

To confirm the direct action of miR-133a in the control of TPM4 expression, we amplified and cloned a 300 bp fragment, containing the putative 3′-UTR responsive element, into the pGL3 vector, replacing a portion of the 3′ UTR of the luciferase gene. The generated vector was used in a luciferase reporter assay (Figure 2B), by using the TMP4-3′ UTR in association with miR-133a or with a scramble sequence. We observed a strong decrease of luciferase activity only when miR-133a was present (Figure 2C), thus demonstrating the direct interaction between miR-133a and the responsive element located in the 3′-UTR of TPM4.

We also isolated exosomes from the plasma of three patients (highest plasma levels of miR-133a), extracted their RNA, and analysed the samples for the presence of miR-133a. The presence of the miR was also detected in exosome samples (Figure 2D), suggesting that miR-133a release relies also on an active secretion mechanism, which could be of regulatory importance. Interestingly the exosomal transfer of miR-133a has been proved, in a system of C2C12 Myocytes and mdx Mice, to be able to significantly decrease cell death and regulate skeletal muscle regeneration in vivo [46].

### 2.3. TPM4 and miR-133a in Myocyte Proliferation and Differentiation 

**Figure 3:**
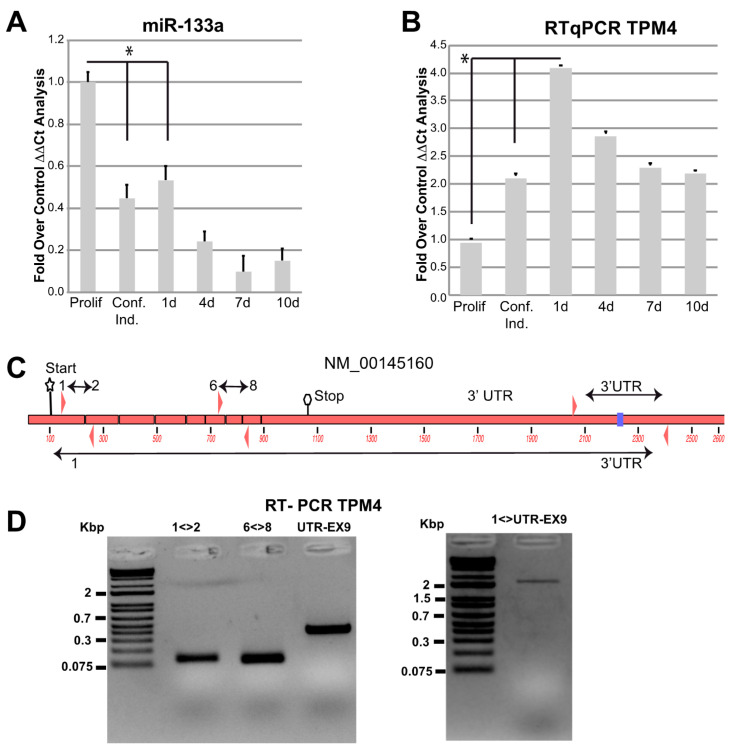
miR-133a and TPM4 expression. miR-133a (**A**) and TPM4 (**B**) expression during myocyte differentiation was analysed by RTqPCR, values were expressed as fold over control. The ΔΔCts were calculated by using housekeeping transcript (actin and GAPDH), and all data were normalised on the proliferating cells condition (*n* = 3, * *p* < 0.01, *t*-test two tails, coupled). (**C**) Structure of TPM4 with primers amplifying different combinations of exons and of the 3′ UTR (NCBI identification number NM_001145160.1). (**D**) Results of the RT-PCR showing the amplification of the full-length transcript including the 3′ UTR (DNA marker: Thermo Scientific™ O’GeneRuler 1 kb Plus DNA Ladder).

To clarify the miR-133a-dependent regulation of the newly identified target TPM4, we used cultured human myocytes from donors. First, we analysed miR-133a and TPM4 expression levels during myocytes differentiation. We extracted RNA from proliferating, confluent, and one day after confluence up to 10 days cells and we checked for miR-133a and TPM4 levels, by qPCR. We observed a downregulation of miR-133a expression early during differentiation (Figure 3A), paralleled by an upregulation of TPM4 expression (Figure 3B).

TPM4 seems to be expressed in different isoforms in muscle [47], so we verified which is the expressed isoform in our experimental conditions, by using four different combinations of primers (Figure 3C). One located in exon 1–2, one in exon 6–8 and the third located in the 3′ UTR, able to amplify the fragment containing the miR-133a responsive element. Moreover, we performed an additional amplification using a forward primer located in exon 1 and a reverse in 3′ UTR, also containing the miR-133a responsive element. The results showed positive amplification for all these combinations (Figure 3E), indicating the presence of a full transcript containing all exons and including the responsive elements in the 3′UTR [47,48,49].

**Figure 4:**
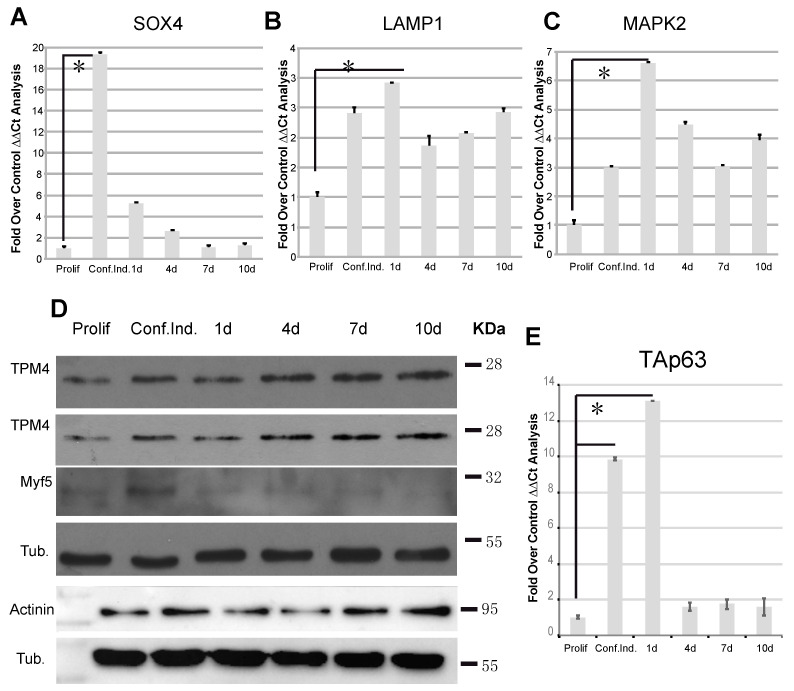
TPM4 and differentiation marker analysis in differentiating myocytes. (**A**) RTqPCR analysis of the differentiation marker SOX4, direct target of miR-133a. The marker shows an inverse expression in respect to miR-133a, confirming the published data. (**B**,**C**) Additionally, LAMP1 and MAPK2 involved in myocyte differentiation show the correct expression; they are upregulated in the time course (**A**–**C** *n* = 3). (**D**) Protein analysis of TPM4, Myf5 and α-actinin, showing TPM4 and α-actinin upregulation and Myf5 downregulation (*n* = 6). (**E**) RTqPCR TAp63 analysis: the transcription factor is expressed in the early phases of differentiation (*n* = 3). The RTqPCR is shown in fold over control. The ΔΔCt was calculated using actin as housekeeping transcript, and all data was normalised on the proliferating cells condition (* *p* < 0.01, *t*-test two tails, coupled).

In order to confirm that our myocytes undergo proper differentiation, we tested the regulation of known genes involved in this process. SOX4, a transcription factor involved in embryonic, cardiac muscular, and nervous development, is increased during myocytes differentiation and it is known that its acetylated form plays a role through SOX4-dependent Cald-1 promoter activation [50]. It has also been demonstrated that miR-133a could physically interact with SOX4 3′UTR, leading to its downregulation [51]. SOX4 overexpression has been described to induce the lysosomal-associated membrane protein 1 (Lamp1) and the mitogen-activated protein kinase 2 (Map2k2) [52], and these two transcripts are highly expressed in differentiated myocytes [52]. Our results showed that, during differentiation, miR-133a decreased expression (Figure 3A) and TPM4 upregulation (Figure 3B), were coupled to SOX4 upregulation (Figure 4A). Accordingly, SOX4 upregulation gives rise to the positive modulation of its direct targets Lamp1 (Figure 4B) and Map2k2 (Figure 4C).

During differentiation, myoblast cells fuse into myotubes leading to the formation of mature muscle fibres, and the process is controlled by many other factors. Among them, Myf5 (proliferating cell marker) expression is upregulated in satellite cells, and progressively decreases when myoblasts exit from the cell cycle and begin to differentiate [53]. In order to confirm the miR-133a-dependent modulation of TPM4, in our proliferation/differentiation model, we analysed proteins extracts belonging to samples at the same time points used for the transcriptional analysis. We observed TPM4 upregulation in two representative tests (Figure 4D, first and second panel), supporting the direct effect of miR-133a on the TPM4 transcript. Furthermore, we observed also a decrease in the Myf5 protein levels at late time points, thus supporting myocyte differentiation (Figure 4D, third panel). Another important player in myocyte differentiation is α-actinin, a member of the spectrin family, which acts as a actin filament cross-linker and as one of the integral Z-disc proteins [54]. High levels of α-actinin protein expression have been described during differentiation of myoblasts into myotubes, coupled to the miR-133a expression decrease [14]. As shown in Figure 4D (lower panels), α-actinin protein expression increased during differentiation, in accordance with the published data. The α-actinin is also important because it is one of the proteins that forms the stress fibres, and its expression can be modified in microfilament readaptation during cell transformation [7].

### 2.4. Immunofluorescence Analysis of TPM4 in Myocyte Differentiation 

**Figure 5:**
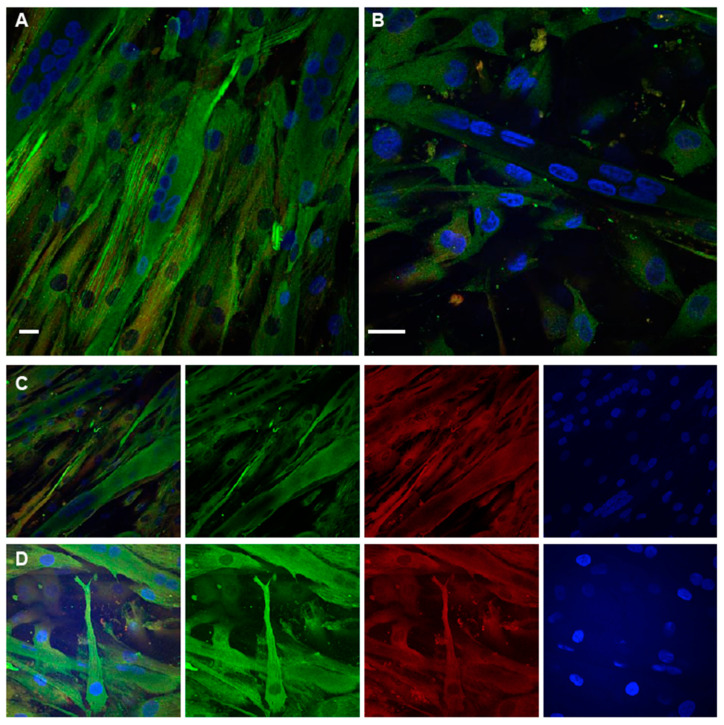
TPM4 and α-actinin staining in differentiating myocytes. (**A**,**B**) Confocal immunofluorescence analysis performed at day 1. In the field are present mono-nucleated cells expressing TPM4 (green) organised in filaments and differentiated myotubes with a lower and less organised expression of the protein. (**C**,**D**) Panels with separate channels showing the concomitant expression of TPM4 (green) and α-actinin (red) in the cytoplasm. Starting from the left first panel is a merged image of all channels, the second panel shows the captured image using only the 488 nm laser (FITC), the third is the capture using the 561 nm laser (TRITC), the last using the 401 nm laser (DAPI, blue). DAPI is used for nuclei staining. Bars = 10 µm.

The immunofluorescence analysis was performed using confocal microscopy (Figure 5A). TPM4 seems (green) to be organised in filaments in single cells and its distribution changes during myotubes formation, as proved by the presence of multinucleated (in blue colour) cells in Figure 5A,B. We also used α-actinin as a co-marker (red) for the differentiation process [14]. The simultaneous detection of TPM4 and α-actinin showed that these two proteins follow the same expression pattern and are localised in the same structures (Figure 5C,D). This result could be relevant to analyse the role of TPM4 in microfilament stress fibres physiology.

### 2.5. TAp63 Controls the Expression of TPM4 in Myocytes and in Colon Cancer Cells

The TAp63 transcription factor has a role in several specialised cell districts [55,56] as well as in myocyte differentiation [57]. For this reason, we checked TAp63 expression in our model system, and the results showed an upregulation of the transcription factor in the passage from proliferating to differentiating cells (Figure 4E).

**Figure 6:**
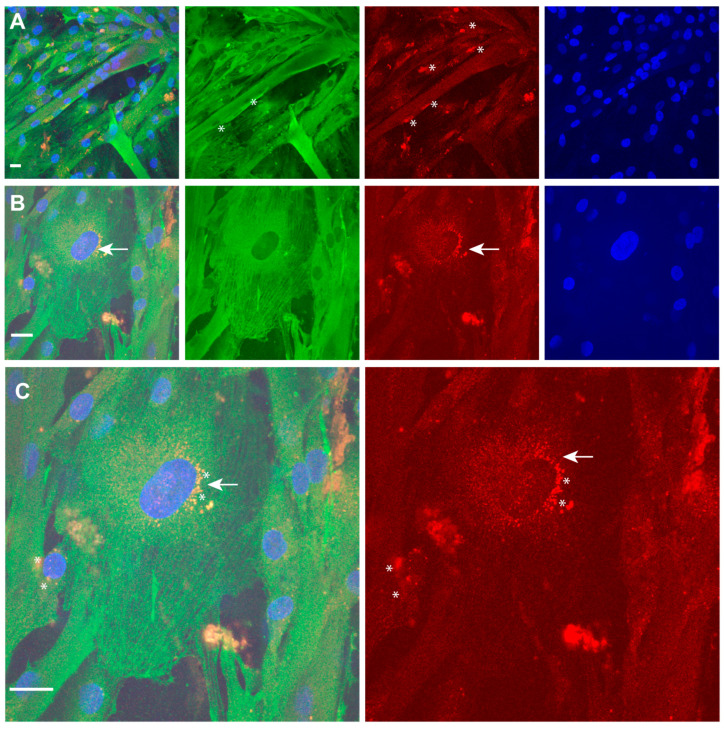
TPM4 and p63 expression in differentiating myocytes. (**A**,**B**) Identification of p63 (red) positive nuclei in differentiating myocytes. Only some mono-nucleated cells (stars) are positive. (**C**) Enlargement of mononucleated cells shows fibrillar distribution of TPM4 (green) and perinuclear localisation of p63 (red), probably identifying the time of the transport from ER to nucleus (arrow and stars). DAPI (blue) was used for nuclei staining. Laser wavelengths as the previous figure. Bars = 10 µm.

We further analysed, by immunofluorescence analysis (Figure 6), the expression pattern of p63 (red) and TPM4 (green) in the early stages of myocyte differentiation and we found that p63 expression is not detectable in differentiated myotubes. The staining for the TAp63 isoform is not a standard procedure, since its expression is limited to cells under the differentiation signalling stimulus. In fact, in our staining, only a few nuclei of isolated cells proved to be positive for p63 (Figure 6A, stars). Interestingly, a positive staining, showing a perinuclear localisation, was observed in cells showing TPM4 organised in filaments, the stage prior to the myotube formation (Figure 6B,C, stars and arrow), probably indicating the transport of the transcription factor from the endoplasmic reticulum to the nucleus.

**Figure 7:**
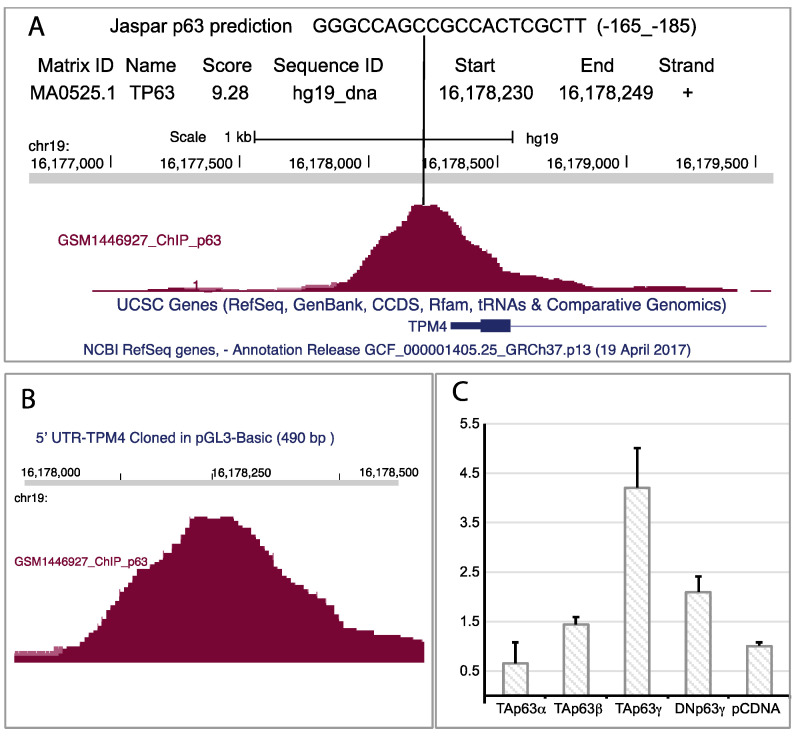
p63 regulatory element on TPM4 promoter. (**A**) Jaspar prediction (sequence in the upper part of the panel (**A**)), and reads counted from RNAseq, suggest the possible presence of a p63 responsive element. Jaspar prediction is obtained by using a specific software that analyses DNA sequence with a mathematical matrix to identify putative transcription factor binding sites. Interestingly, the “in silico” analysis and the RNA-Seq return the same site in TPM4 5′ UTR. (**B**) Enlargement of the genomic region containing the reads from the RNA-Seq. (**C**) Luc-Assay using a 490 base pair region of the promoter containing the responsive element, showing that the TAp63γ isoform nicely regulates the TPM4 promoter (*n* = 4).

We speculated that TAp63 could drive TPM4 expression. The analysis of the TPM4 promoter sequence, made by using the UCSC Genome browser, and the data from a published ChipSeq [58], showed accumulation of reads in a region located at −175 base pairs from the ATG, in the 5′UTR of the gene (Figure 7A,B), suggesting the presence of a p63 responsive element. This analysis perfectly correlates with the “in silico” Jaspar p63 prediction promoter analysis, that shows the presence of the sequence GGGCCAGCCGCCACTCGCTT, located at position −185 to −165, as a putative p63 binding site. To verify whether this was true, we cloned a 490 base pair region containing the responsive element into the PGL3 vector, and we performed a luciferase assay. The obtained results showed that, among the different p63 isoforms, only TAp63g results in a strong activation of the promoter (Figure 7C), demonstrating that this transcription factor is able to directly transactivate TPM4.

**Figure 8:**
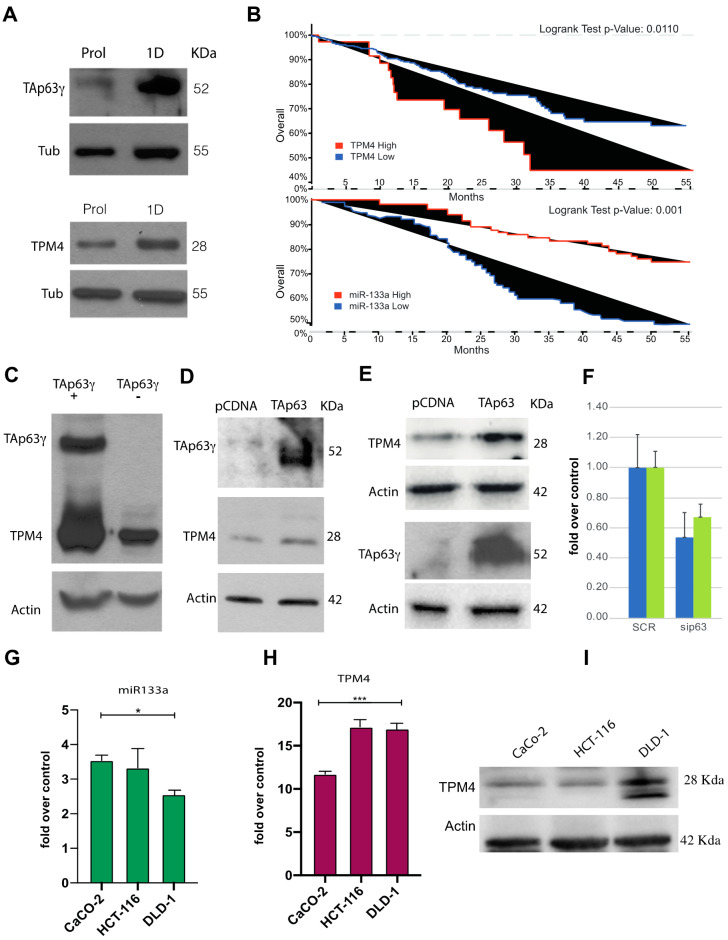
In vivo transactivation of TPM4 by TAp63γ, and overall-survival analysis of a CRC patient cohort. (**A**) TAp63γ (approx. 50 KDa) expression increases during early differentiation of myocytes, and TPM4 expression follows the same kinetic, “Prol” indicates the proliferating myocytes, “1D” indicated myocytes at 1 day of differentiation. (**B**) Upper panel, analysis, performed by using cBioportal, on colorectal adenocarcinoma (TCGA, PanCancer Atlas) showed higher survival for patients with low TPM4 expression (Logrank Test *p*-Value: 0.011; *p*-Value = 0.0190, *q*-Value = 0.0379). Lower panel, data reanalysed from Wang et al. [59], showing the inverse correlation with survival of miR-133a respect to TPM4. (**C**) TAp63γ is able to drive the expression of TPM4 also in H1299 tumour cells, demonstrating the presence of TAp63γ- TPM4 regulation also in cancer cells. (**D**) Upregulation of TPM4 protein expression by the ectopic expression of TAp63γ in caco-2 cells. (**E**) Upregulation of TPM4 protein expression by TAp63γ in HCT-116 cells, showing that the transcription factor is able to drive the transcription of TPM4 also in these cells. (**F**) RTqPCR, showing downregulation of TPM4 after inhibition (siRNA) of p63 in HCT-116 (blue) and Caco-2 (green, *n* = 3) cells, demonstrating that the regulatory pathway is TAp63 specific and valid also at protein level. (**G**) qPCRT amplification of miR-133a in CaCo-2, HCT-116 and DLD-1 (*n* = 3; * *p* < 0.05, *t*-test two tails, coupled). (**H**) mRNA TPM4 expression in CaCo-2, HCT-116 and DLD-1 (*n* = 3). (I) WB showing TPM4 expression in CaCo-2, HCT-116 and DLD-1 (*n* = 3; *** *p* < 0.01, *t*-test two tails, coupled).

Further, we analysed TAp63γ and TPM4 protein expression during the initial stages of myocyte differentiation (Figure 8A). Our results suggest similar kinetics for the two proteins during the passage from proliferating to differentiated (1 day of differentiation) cells, thus demonstrating that this pathway is active in vivo. Moreover, we also observed a downregulation of TPM4 mRNA level after p63 silencing with siRNA (Figure 8F).

Both TAp63 and miR-133a can regulate TPM4 expression. Recent literature data connect physical exercise and colon cancer (CRC) pathogenesis, as well as report that miR-133a levels are significantly linked to tumour differentiation, invasion, metastasis and clinical TNM stage in CRC patients [59]. The decrease of this miR could be related with the disease progression since it is considered a tumour suppressor microRNA. For this reason, we further investigated the miR-133a, TPM4 and TAp63, in CRC. Indeed, actin-TPM4 microfilaments are involved in contraction, cell morphology, cell motility and intracellular vesicle transport, recent evidence also suggested its crucial role in tumour development. In fact, the transformed cell phenotype, characterised by rearrangement of cytoskeleton microfilaments, morphological modifications and increased cell motility, is often associated to an increased expression of TPM4 isoforms [8,9,60]. We analysed public microarray data, belonging to a cohort of 683 patients (cbioportal). Our results show a correlation between TPM4 expression and the overall survival of patients (Figure 8B, upper panel), patients with increased TPM4 expression have a low overall survival. This observation is in accordance with literature data reporting that the increased expression of TPM4 is able to enhance cell migration while its downregulation shows suppressive properties [8]. Based on this evidence, we investigated TAp63 expression as well as miR-133a. The first interesting line of evidence was obtained by reanalysing published data [59] about miR-133a expression in CRC patients. The results show that the expression of miR-133a in terms of survival (Figure 8B, lower panel) perfectly correlate with TPM4 expression. In this case, a higher expression of the miR (low expression of TPM4) correlates with longer survival, thus confirming our hypothesis.

Our data on muscle cells show that TAp63 drive the TPM4 expression. To verify this in tumour development, we overexpressed TAp63 in different tumour cell lines, H1299 (lung cancer, Figure 8C), CaCo-2 (colon adenocarcinoma, Figure 8D) and HCT-116 (colorectal carcinoma, Figure 8E).

In all tumour cell lines, only a small amount of TAp63 is detectable in steady-state level, in accordance with its tumour suppressor role, even if the TAp63 ectopic expression retains the possibility to drive the TPM4 expression. 

The Caco-2 cells are not characterised as metastatic cells, they show a well differentiated phenotype, HCT-116 cells are able to form tumours when inoculated in BALB/Nude mice, while DLD-1 show positivity to vimentin staining (Supplementary Figure S3) representing the most aggressive colon cancer cells, able to form distant metastasis. DLD-1 showed the higher downregulation of miR-133a expression. This miRNA is less expressed in DLD-1, HCT-116 rather than CaCo-2 tumour cell lines (Figure 8G), and accordingly higher TPM4 mRNA levels were found in DLD-1 rather than in CaCo-2 (Figure 8H). At protein level, it is clear the upregulation of TPM4 in response to miR-133a downregulation, in accordance with the tumoral aggressiveness of cell lines, thus confirming that this miRNA is important for TPM4 regulation also outside muscle differentiation. 

From literature, miR-133a can inhibit cell growth, migration, metastasis by targeting LASP1 in colorectal cancer [61]. Furthermore miR-133a is able to repress gastric cancer growth and metastasis [62] as well as in breast cancer [63]. 

We found downregulation of miR-133a in metastatic DLD-1 and HCT-116 cells which shown an aggressive behaviour. TPM4 follow the exact inverse kinetics demonstrating that miR-133a represses TPM4 in this system. 

**Figure 9:**
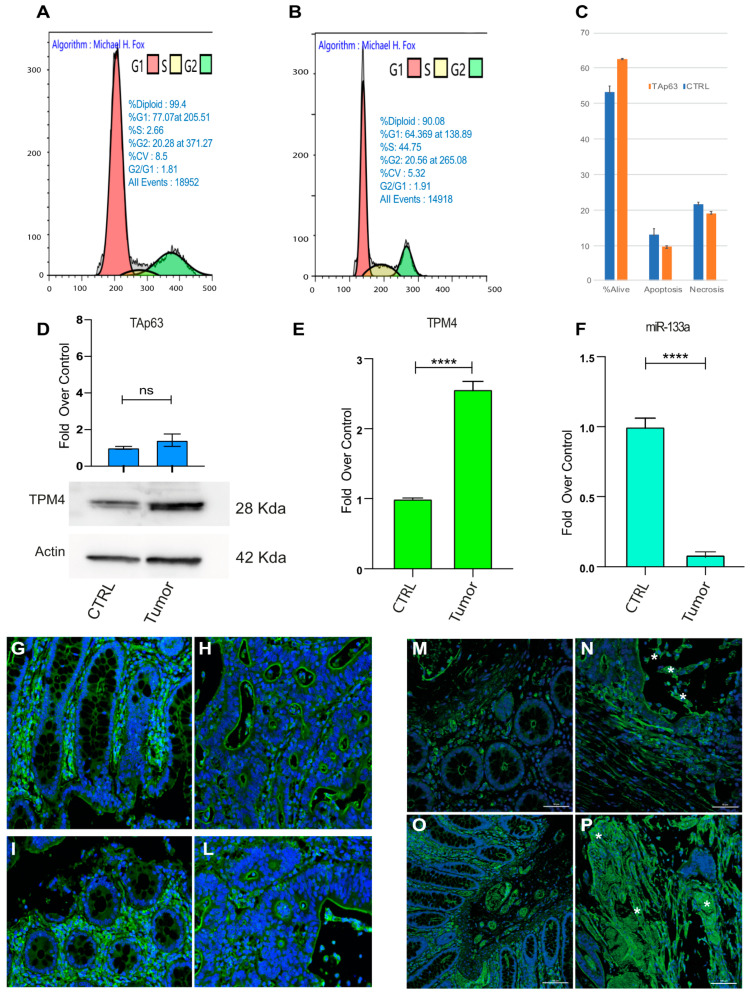
Cell cycle analysis and TPM4 expression in CRC patient specimens. (**A**) Cell cycle analysis in HCT-116 CRC cells expressing TAp63γ (**B**) control cells. Representative experiments are shown (*n* = 3). (**C**) Vitality analysis showing that TAp63 expression does not alter the apoptotic program of these cells. (**D**) PCR of TAp63 in control and CRC tissue (**top**) TPM4 Western blot in normal and CRC tissues (**Low**). (**E**) TPM4 expression level in control/CRC tissues. (**F**) miR-133a expression level comparison between colon tissue and colon cancer tissue (**** *p* < 0.001, *t*-test two tails, coupled) (**G**,**P**) representative sections from two patients. (**G**,**I**) show the tumour adjacent normal area of the mucosa, in which is recognised the presence of regular glands, monolayered with a large luminal vacuole containing mucus, whose cytoplasm is TPM4 stained (green). In (**H**,**L**), a contiguous neoplastic area (adenocarcinoma) is represented, consisting of irregular glands, multi-layered, free of mucus, arranged back to back. TPM4 is present in the wall of the mucosal vessels (DAPI, blue, identify the nuclei). (**M**–**O**) Tumour adjacent normal area of mucosa, (**N**–**P**) Neoplastic area characterised by a high detection of TPM4 (green). In particular, adenocarcinoma cells recognisable by typical morphology, showing high cytoplasmic TPM4 staining (stars).

Moreover, cell cycle analysis of cell HCT-116 lines transfected with TAp63γ isoform demonstrated that there is an accumulation of cells in the G1 phase (Figure 9A), as compared to the mock transfected ones. Furthermore, TAp63 expression slightly decreases the percentage of apoptotic or necrotic cells (Figure 9C).

To deeply analyse the role of miR-133a, TPM4 and TAp63 in vivo, we used human sample tissues, from patients affected by metastatic CRC. CRC tissues showed higher protein levels of TPM4 than normal adjacent tissues (Figure 9D, low panel) and low levels of mRNA (Figure 9E) were also revealed. Consistently, miR-133a results to be less expressed in colon cancer tissue rather than in the normal tissues (Figure 9F), thus demonstrating the connection between the loss of miR-133a during tumour development, with the TPM4 upregulation. Moreover, the investigation on the TAp63 levels showed also a slight upregulation of this transcription factor, indicating that the latter probably does not participate in the upregulation of TPM4. 

Tissue sections, belonging to specimens of high-grade colon adenocarcinoma (pT3, pT4a, pT4b) and showing the normal mucosa and the infiltrating tumour, were stained for TMP4. Figure 9G–P shows a representative image from a sample of a patients with lymph node metastasis (see supplementary Figure S4 for sections of six different patients and a table reporting the characteristics of each one Table S1). In the pre-cancerous tissue TPM4 is expressed in organised fibres clearly detectable in normal cells (Figure 9G,I,M,O) and regular glands are recognised. This architecture is lost in the tumoral tissue invading the mucosa and generating the metastasis (Figure 9H,L). Nevertheless, in the irregular area of CRC tissues, an increase of TPM4 (green), in disorganised fibres, was found in the cytoplasm of cancerous cells characterised by altered morphology (Figure 9N–P, stars). In these sections TPM4 organised fibres staining is only detectable in the vascular tissue, since TPM4 is present also in the smooth muscle of arterioles of the neo vascularised tumour region. In supplemental data, the staining for TPM4 in six independent patients is shown confirming these results.

## 3. Discussion

The classical physiological function of tropomyosins has been defined by considering their role in muscle contraction [64]. At the structural level, tropomyosins are proteins that possess a dimeric a-helical coiled-coil structure, along the entire length. Though they seem to show a quite simple protein structure, molecular and genetic studies showed an important complexity in the functions exerted by tropomyosins among different organisms. Moreover, it has been shown that different tropomyosin isoforms carry specific properties and are important in different cellular functions, including regulation of cell signalling and differentiation [65,66]. In malignant cells, the cytoskeleton is reorganised [3], and reprogramming of its composition and structure support tumour cell survival, growth, and allow the invasion of other tissues [4].

On the basis of the identified downregulation of high molecular weight tropomyosins, upon cell transformation, and its association with the loss of the stress fibres, tropomyosins have been proposed as biomarkers of cell transformation and cancer, for a long time [67]. Recent studies demonstrated a direct link between EMT and actin dynamics, showing a significant role for cytoskeleton and microfilaments in the pathophysiology of tumour generation and progression [68,69].

TPM4 has been associated with inflammatory myofibroblastic tumour and autosomal dominant macrothrombocytopenia (prevalence: <1/1,000,000 worldwide), even if in this case, the association is due to the presence of TPM4-ALK and ALK-TPM3 translocations, resulting in the formation of a fusion gene and transcript, leading to a function different from the physiological TPM4 function [70]. It has been also shown that deletion/truncation involving the TPM4.2 transcript influences the maturation of megakaryocytes, leading to a rare form of platelet disease [71].

Recent evidence correlated TPM4 expression with cancer development, specifically with neoplastic cell invasion and metastatisation [8,9,60]. The ability of TPM4 to promote cell motility and migration is reported in lung cancer [8], and it has been known that TPM4 overexpression in HCC tissues aggravated the malignancy of hepatocarcinoma through the negative regulation of SUD-2 [9]. 

Our results demonstrated that colon carcinoma patients showing higher TPM4 expression had a lower survival, as compared to those with lower TPM4 expression. Moreover, by using our model of skeletal muscle differentiation, we were able to identify miR-133a as the miRNA able to negatively modulate TPM4 expression. This result, within the data showing that CRC patients with high expression of miR-133a show a longer survival [59], confirms the relationship between these two effectors in CRC physiology. 

Most importantly, the downregulation of miR-133a in CRC tumour tissues has been associated with the development of distant metastasis, advanced Dukes and TNM staging, and poor survival. In fact, it has been reported that miR-133a is able to reduce cell viability in HCT-116 and DLD-1 as well as miR inhibited cell growth in nude mice through G0-G1 phase arrest [72]. Moreover miR-133a re-expression exerted a tumour suppressor role by interacting with RFFL, upregulating p53 and its downstream effector p21 in a colon cancer cell line [72]. Furthermore, Wang et al. reported that miR-133a was able to repress metastasis through MAPK pathway inhibition by targeting LIM [61]. 

We propose here that miR-133a tumour suppressor role could be related also to the miR-133a-dependent regulation of TPM4 expression.

**Figure 10:**
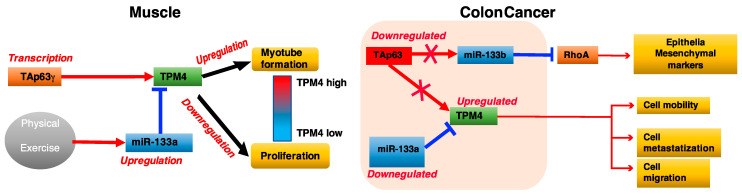
Cartoon explaining the mechanism of interaction between TAp63, miR133, and TPM4 in muscle and colon carcinoma.

Our results demonstrated that TAp63γ, the gamma isoform of TAp63, showing the higher transcriptional activity [73], is able to regulate TPM4 expression in muscular district (Figure 8), but not in CRC. We propose a dual role for miR-133a in muscle growth and differentiation as well as in cancer development and progression, in which TPM4 has a central role (Figure 10).

The miR-133a→TPM4 and TAp63γ →TPM4 axes are key elements in the muscular process. In fact, miR-133a downregulation removes an inhibitory signal, and Tap63γ expression synergistically leads to TPM4 expression, leading to the differentiation of muscle cells. Conversely, high miR-133a expression levels, in response to training, results in a decreased expression of TPM4 and in the induction of muscle cells proliferation. In colon cancer the miR-133a-TPM4 axis is able to control cell migration and invasion by remodelling of the cytoskeleton. In these settings, low level of miR-133a observed in CRC results in the increase of TPM4 expression levels responsible for the modification of the cytoskeleton architecture, high cell motility, migration and metastatisation, possibly favouring EMT. Moreover, growth-inhibitory function of miR-133a in colon cancer cells has been reported, therefore its tumour suppressor role. In particularly, the transfection of this miR increases the G_O_-G_1_ arrest through p53/miR-133a–mediated induction of p21 [72] and reduces significantly the cell viability, migration and invasion by targeting eIF4A1 in HCT-116 cells [74]. MiR-133a is also able to inhibit cellular proliferation and colony formation by targeting SP1 protein that binds to IGF1-R promoter blocking IGF-1 involved in colon cancer progression [75].

It was previously known that miR-133a was a specific regulator of skeletal muscle differentiation and proliferation [13,14], and we found that during physical exercise miR-133a is augmented in the plasma of training subjects, resulting in the downregulation of TPM4 in plasma. The same happens at cellular level.

Our results identified TPM4 as a new candidate gene in the physiology of colon cancers, suggesting that the miR-133a-TPM4 axis plays a crucial role in tumour development. The decrease of miR-133a and the increase of TPM4 expression levels could be relevant to the metastatic process and highlight a key role for both miRNA and protein, as also shown from the survival data with respect to the expression level of both TPM4 and miR-133a in CRC patients. The role of TAp63 should be further investigated, since we demonstrated that the gamma variant is able to regulate TPM4, but in vivo it is not possible to distinguish between the 3′ variants (α, β, γ, δ, ε). The data with respect to the expression of the TAp63 isoform in CRC tissue leads to consider the axis miR-133a-TPM4 as the major effector in cancer. miR-133a has been proven to be an upstream effector of this process, leading also to the possibility to use skeletal muscular differentiation to discover effects of microfilament remodelling in cancer cells.

Compounds able to target TPMs have been used to kill cancer cells, via shifting the G:F actin ratio inducing apoptosis induction [76], and their ability to synergise with other anti-microtubule drugs [76]. This suggests the possibility to use TPM4 targeting compounds, stabilising its presence in microfilaments, as a possible therapeutic target in chemotherapy.

Finally, the mechanism proposed is that the downregulation of miR-133a in CRC leads to TPM4 overexpression, modifying the cell cytoskeleton, increasing the invasiveness of the tumour and associating with a poor prognosis. These results add data to the interesting question about the link between physical activity and protection against colorectal cancer. For this reason, understanding the role of TPM4 in muscular district could give insight in future CRC studies.

## 4. Materials and Methods

### 4.1. Probands

For this study, we used 22 male subjects from a previous study [43] performed at the University of Tor Vergata spanning from 22 to 32 years old. The subjects were students of BSc degree in Sport Sciences at the University of Rome Tor Vergata and from a national level of five-a-side football players. The subjects participated in a training schedule aimed at the development of the maximum dynamic strength by overloads with an additional electrostimulation.

### 4.2. Blood Sampling

Blood samples were taken using BD Vacutainer (K2E 5.4 mg), 3 mL tube, plasma was recovered after centrifugation and a two step centrifugation protocol was used [44] to avoid platelet contamination. The venepuncture was performed before the exercise (T0), 20 min after the end of the exercise, as well at 48 and 72 h after the end of the exercise.

### 4.3. MicroRNA Extraction from Plasma Samples

The extraction of microRNA from plasma and exosomes was performed by Maxwell 16 Instrument (AS3000, Promega, Madison, WI, USA) using Maxwell 16 miRNA Tissue Kit (AS1470, Promega, Madison, WI, USA) according to a modified manufacturer’s protocol, created by Promega to be used in this instrument instead of the RSC version. In total, 400 µL of plasma was used. To prepare samples, plasma was homogenised with Homogenization Solution/1-Thioglycerol (1:50) and then incubated at 37 °C for 15 min in an equal volume of Lysis Buffer (MC501C) with the addition of Proteinase K Solution (MC5005C).

### 4.4. Exosome Isolation from Plasma

To isolate exosomes, plasma EDTA samples were obtained from probands and centrifuged twice for 10 min at room temperature (according to reference [44]). In total, 400 µL of supernatant (plasma) was suspended in PBS 1X (0.5 volumes) on ice. Then, 0.2 volumes of total exosome precipitation reagent (Invitrogen) were added to each sample and vortexed until the solution was homogeneous. Subsequently, samples were incubated at room temperature for 10 min and centrifuged at 10,000× *g* for 5 min. The pellet containing exosomes was resuspended in PBS 1X and stored at −20 °C until use.

### 4.5. mRNA and miRNA Extraction from Cells

The extraction of mRNA and miRNA from myocytes was performed by mirVana^TM^ miRNA isolation kit (Thermo Fisher Scientific, Waltham, MA, USA) according to manufacturer’s protocol. Total RNA was extracted from colon cancer cells using TRIzol^TM^ Reagent (Thermo Fisher Scientific, Waltham, MA, USA) according to manufacturer’s protocol.

### 4.6. Protein Extraction

After removing culture medium, lysis buffer 10X pH 7.7 (50mM TRIS, 150 Mm NaCl, 10 mM EDTA, 1% Triton) was added to myocytes. Cells were scraped and after incubation for 30 min at 4 °C, lysates were centrifuged at 10,000× *g* for 20 min at 4 °C. Proteins were contained in the supernatant and stored at −20 °C until use. HCT-116 and CACO-2 cells were lysed using RIPA buffer (Tris-HCl 50mM pH 7.5, NP40 0.5%, Deoxycholate 0.5%, SDS 0.1%, NaCl 250 mM, EDTA 1mM).

### 4.7. RT and Real Time PCR

mRNA and microRNA, extracted from plasma exosomes, myocytes and colon cancer cells, were converted in cDNA using different protocols. For microRNA, TaqMan ^tm^ MicroRNA Reverse Transcription Kit (Applied biosystems, Waltham, MA, USA) with the addition of specific primers for each microRNA (mir-1, mir-133a mir-206 and U6) was used, while for mRNA, Goscript ^tm^ reverse transcription system (Promega) was used. To analyse gene expression levels, GoTaq ^®^ pcr Master Mix (Promega) was utilised. Real-Time primers were TPM4 forward 5′-CTGAAATCTCTGGAGGCTGC-3′, reverse 5′-AAATTCAGCACGGGTCTCAG-3′, SOX4 forward 5′-ACCGGGACCTGGATTTTAAC-3′, reverse 5′-AAACCAGGTTGGAGATGCTG-3′, LAMP1 forward 5′-AGGCTTTCAAGGTGGAAGGT-3′, reverse ATGAGGACGATGAGGACCAG, ACTIN forward 5′-GTTGCTATCCAGGCTGTGCTA-3′, reverse 5′-AATGTCACGCACGATTTCCCG-3′, GAPDH forward 5′-GCCCAATACGACCAAATC-3′ reverse 5′-AGCCACATCGCTCAGACA-3′, MAP2K2 forward 5′- GTGCTGAAAGAGGCCAAGAG-3′, reverse 5′ TGCATGATCTGGTGCTTCTC-3′, ACTIN forward 5′-GTTGCTATCCAGGCTGTGCTA-3′, reverse 5′-AATGTCACGCACGATTTCCCG-3′. For HCT-116 cells, reverse transcription was performed using SensiFAST^TM^ cDNA Synthesis kit and GoTaq ^®^ pcr Master Mix (Promega) for Real-Time PCR, according to manufacturer’s protocol. Primers:

TAp63: forward 5′-GGACTGTATCCGCATGCAG-3′, reverse 5′-GAGCTGGGCTGTGCGTAG- 3′ TPM4 forward 5′-CTGAAATCTCTGGAGGCTGC-3′, reverse 5′-AAATTCAGCACGGGTCTCAG-3′. For miRNA level analysis, TaqMan^®^ Universal PCR Master Mix No AmpErase ^®^ UNG (Applied Biosystems, Italy) was used with the addition of a specific probe for each miRNA (probe for miR-1, miR-133a, miR-206 and probe for U6 from Applied Biosystems). Real time amplification was performed using 7500 Real Time PCR System (Applied Biosystems) and data analysis was completed using 7500 Software v2.3, using actin and U6 for normalisation.

### 4.8. Cloning and Luc-Assay

To check the interaction between miR-133a and its putative binding site located in 3′-UTR of TPM4, H1299 cells were co-transfected with premiR-133a and pGL3 vector that contained cloned 3′UTR of TPM4. Subsequently, luciferase assay was performed by Dual-Luciferase Reporter Assay System (Promega) according to previously established protocols [77]. 3′UTR region was amplified using specific primers through PCR (forward 5′-GTCTAGATCAGGTCAGCCAT-3′, reverse 5′-GATCTAGACCAGAAGCAGGGTG-3′). Purified PCR products and pGL3 vector were incubated with XBAI restriction enzyme (20,000 U/mL, New England BioLabs) that recognises T/CTAGA restriction sites. Ligation step was performed by Quick T4 DNA Ligase. pGL3 vector + 3′UTR was amplified using competent TopTen bacteria cells. pGL3 vector contains the gene that confers ampicillin resistance necessary for selecting bacteria of interest and the luciferase gene. pGL3 vector + 3′UTR was isolated, purified and sequenced to assess the correct orientation of the 3′UTR fragment.

H1299 cell line is derived from lung carcinoma cells and was used as recipient for luciferase assay, according the producer suggestions (Promega, Madison, WA, USA). The cells were maintained in RPMI Medium (ThermoFisher) supplemented with 10% FBS (foetal bovine serum) and penicillin-streptomycin at 37 °C, 5% CO_2_. When cells were grown up to ~85% of confluence, they were co-transfected using Lipofectamine 2000 (Thermo Fisher Scientific) according to the standard protocol.

### 4.9. Isolation and Culture of Primary Human Muscle Cells

Primary cultures of human muscle cells were initiated from satellite cells of portions of diagnostic muscle biopsies of healthy probands with high-energy femoral fracture. These cells can be obtained using muscular tissue resected in surgical fracture treatment. Exclusion criteria were history of cancer, myopathies or other neuromuscular diseases or chronic administration of corticosteroid for autoimmune diseases (more than 1 month), diabetes, alcohol abuse, and Hepatitis B virus, Human Immunodeficiency Virus or Hepatitis C virus infections. 

Aneural muscle cultures were established in monolayer according to Askanas V protocols [78]. The muscle biopsies were cleaned and transferred to culture dishes with conditioning media2, added with 25% of human plasma and incubated for 7 days checking for fibroblast outgrowth. The tissue was cut into 1 mm pieces and then placed in gelatine-plasma coated dishes with aneural F14 media3 for 5–7 days. After substantial growth, the tissue was removed. The satellite cells were placed on a new dish with 0.2% gelatine. When confluent cultures just started to fuse into myotubes, they were rinsed with Phosphate Balanced Solution *w/o* Ca^2+^ and Mg^2+^ and then switched in F14 medium (Biowest, Nuaillé, FR) containing 5% FBS, PSF, PS and insulin without growing factor. The cultures of myotubes were fed twice a week until use for experimental procedures.

### 4.10. Culture Condition of Colon Cancer Cells

HCT-116 cell lines, derived from human colorectal carcinoma, were purchased by ATCC. Cells were maintained in McCoys’ 5a Medium Modified, Catalog No. 30-2007, with the addition of foetal bovine serum to a final concentration of 10%, and 1% penicillin-streptomycin.

CACO-2 cell lines, derived from human adenocarcinoma, were purchased by ATCC. Cells were maintained in Eagle’s Minimum Essential Medium (EMEM, ATCC 30-2003), with the addition of foetal bovine serum to a final concentration of 10%, and 1% penicillin-streptomycin. 

DLD-1 cell lines, derived from human adenocarcinoma, were purchased by ATCC. Cells were maintained in RPMI Medium (Thermofisher Scientific), with the addition of foetal bovine serum to a final concentration of 10%, and 1% penicillin-streptomycin. Cells were incubated at 37 °C, CO_2_ 5%.

### 4.11. Cell Transfection

Cells were transfected at 60–80% of confluency. For silencing experiments Lipofectamine-RNAiMAX (Invitrogen, USA) was used, siRNA#2 for p63 (Sigma, St. Louis, MO, USA) and scramble as a control (Sigma, lot. WDAA1199), RNAimax/siRNA (2:1), according to manufacturer’s protocol. Cells were harvested after 72 h.

For overexpression experiments, 300,000/dish 60 mm plated cells were transfected used Effectene Transfection reagent (Qiagen, Germany) pCDNA 3.1-HA-T∆p63γ vector and pCDNA 3.1 HA empty vector (Invitrogen, Waltham, MA, USA) as a control, according to manufacturer’s protocol (2 µg plasmid/25 µL effectene reagent). Cells were harvested after 24 h.

### 4.12. Cell Cycle Analysis

Harvested T∆p63γ overexpressing-cells (72 h post transfection) were fixed with 1:1 PBS and methanol-acetone (4:1 (*v/v*) solution at −20 °C), incubated with RNaseA (13 Kunitz/mL) at 37 °C for 15 min and stained with 100 mg/L propidium iodide (Sigma), overnight at 4 °C. Analysis was performed using Beckman Coulter CytoFLEX with CytExpert software.

### 4.13. Immunofluorescence Analysis

Sections from specimens of CRC patients were used for TPM4 analysis. All the patients have had a CRC diagnosis of high grade with resection of tumour, as reported in Supplementary Table S1, and Figure S1.

Human myocytes were fixed in formalin at room temperature for 30 min, permeabilised with Triton 0.1% for 10 min and incubated in blocking buffer (2% goat serum in PBS 1X) for 1 h. Human colon cancer tissues were deparaffinised, washed 3 times in BioClear (Bio-Optica) and rehydrated in solutions with decreasing concentrations of alcohol and increasing concentrations of water. Antigen retrieval was achieved by microwaving the sections in 0.01 M sodium citrate (pH 6.0). Sections were incubated in blocking buffer (PBS1X + 5% goat serum) for 2 h. The primary antibodies used for detection were rabbit polyclonal anti-TPM4 (ThermoFisher, dilution 1:250), mouse monoclonal anti-α-actinin (BM75.2, ab11008 Abcam, dilution 1:100) and mouse monoclonal anti-p63 (ab735, Abcam, dilution 1: 200). The following secondary antibodies were Alexa Fluor 488 goat anti-rabbit IgG (H + L) antibody and Alexa Fluor 568 goat anti-mouse IgG (H + L) antibody (Invitrogen, Carlsbad, CA, USA, dilution 1:1000). Nuclei were stained with DAPI (dilution 1:1000). Cells were mounted using the Prolong Antifade kit (Invitrogen) and analysed with a confocal laser microscope (NIKON Eclipse Ti). Detection of the signal was performed using NIS elements AR4.00.04 software (Nikon, Tokyo, Japan).

### 4.14. Western Blotting

Protein lysates of myocytes and colon cancer cells were resolved on SDS polyacrylamide gels and blotted onto a Hybond PVDF membrane (G&E Healthcare, Waukesha, WI, USA).

Primary antibodies: mouse monoclonal ἀ-actinin (BM75.2, ab11008 Abcam, dilution 1:1000), rabbit polyclonal anti-MYF5 (C-20, Santa Cruz SC302, dilution 1:100), rabbit polyclonal anti-TPM4 (ThermoFisher, dilution 1:1000), mouse monoclonal anti-HA.11 (MMS-101P, Covance, dilution 1:500), mouse anti-T∆p63 (3.1, Borek, dilution 1:250), mouse monoclonal anti-β-tubulin (T7816, sigma, dilution 1:10,000), mouse monoclonal anti-β-actin (A5441 Sigma, dilution 1:50,000).

All antibodies were prepared in blocking solution (milk 5% in PBS 0.1% TWEEN) except primary antibodies for Actin and Tubulin, prepared in PBS 0.1%TWEEN. β-tubulin and β-actin were used as loading control, respectively, for myocytes and colon cancer cells.

### 4.15. Statistical Analysis

The logrank test (Mantel–Cox test) was performed automatically from cbioportal website (total patients = 683, *q*-value 0.039). All statistical analyses were performed using Microsoft Excel program, *t*-test, two tails, equal variance, with *p* < 0.01. The number of experiment and/or sample size are indicated in figure captions.

## Data Availability

Data are entirely presented into the manuscript.

## Supplementary Materials

The following are available online at https://www.mdpi.com/article/10.3390/ijms22189818/s1.
