# Supplementary material for: The miR-133a, TPM4 and TAp63γ Role in Myocyte Differentiation Microfilament Remodelling and Colon Cancer Progression
Source: Int J Mol Sci. 2021 Sep 10;22(18):9818. doi: 10.3390/ijms22189818 (PMC8472330; doi:10.3390/ijms22189818)
Supplement: Supplementary file 1 [file ijms-22-09818-s001.zip › ijms-1327089-supplementary.pdf]

Supplementary Materials

Table S1: Clinical characteristics of colon cancer patients according to TNM classification of tumor.

Number	Age	Sex	Histological Diagnosis	pT grade	Limph Node Metastasis
**6330**	77	M	High grade adenocarcinoma	pT3	N0
**5597**	82	F	High grade adenocarcinoma	pT4a	N2
**4845**	85	F	Low grade adenocarcinoma	pT4a	N0
**11080**	60	M	High grade adenocarcinoma	pT3	N2
**1321**	83	F	High grade adenocarcinoma	pT4a	N2
**3069**	84	M	High grade adenocarcinoma	pT4b	N1

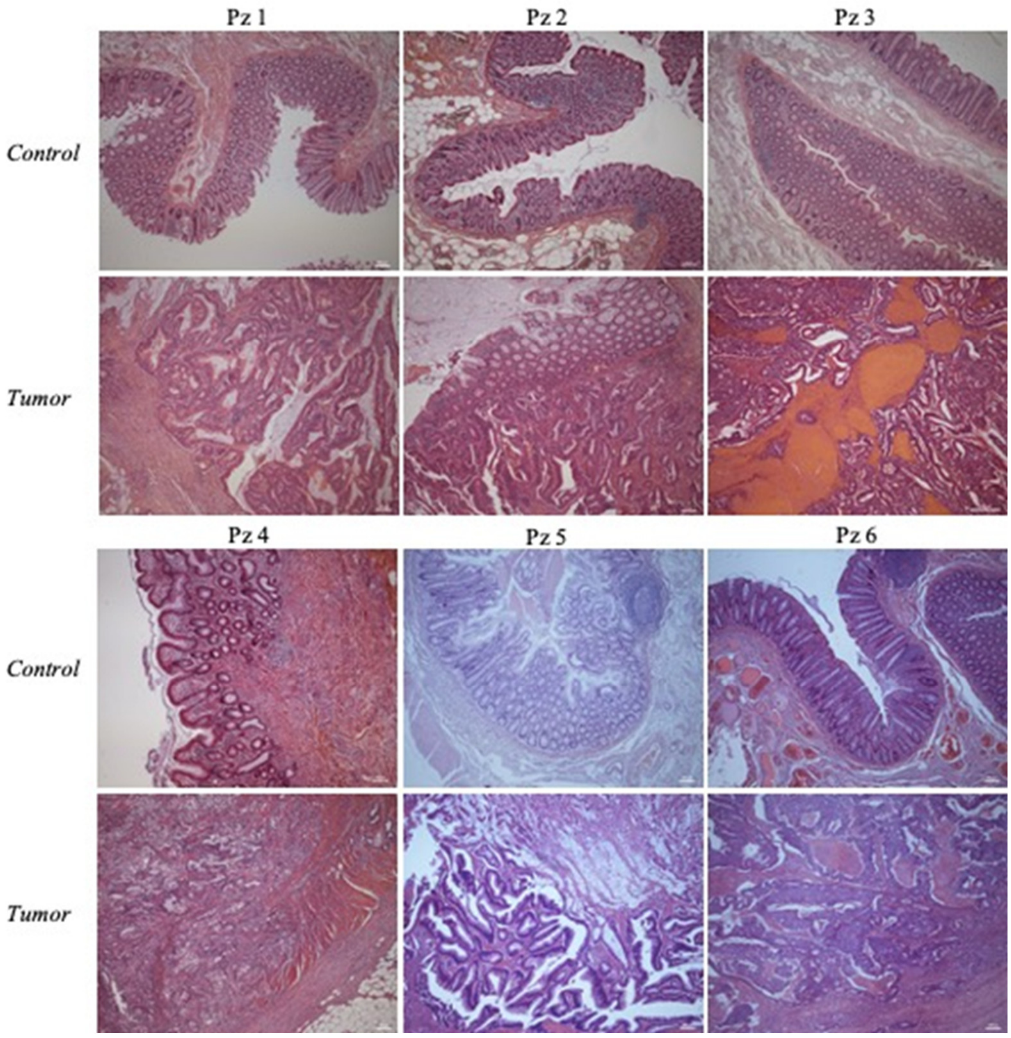

Figure S1. Light microscopy of CRC tissues. Hematoxylin-Eosin staining of adjacent normal area (upper panel) compared with colon cancer tissue (lower panel) for each patient. The staining underlines the morphological alterations consisting of irregular glands, multi-layered.

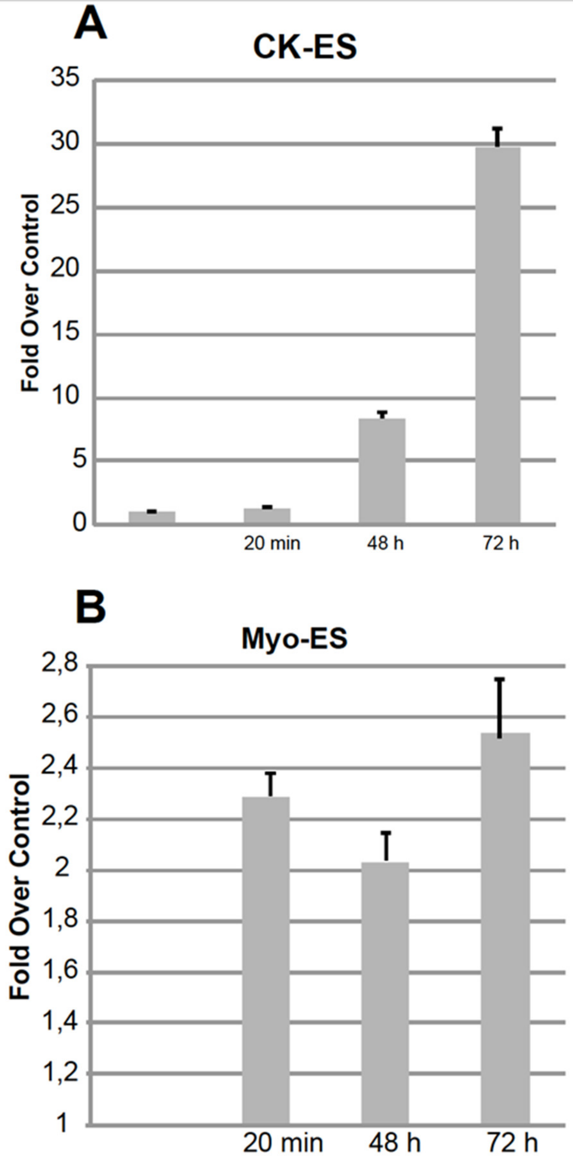

Figure S2. Creatin Kinase and Myoglobin levels. Blood parameters have been measured collecting blood immediately before the training session (T0), after 20 min, at 48 and 72 h. Panel (**A**) shows the increase of Creatin Kinase after training with a maximum detectable peak at 72 h. Panel (**B**) represents the trend of Myoglobin levels after physical exercise. Also for this marker, the maximum detectable peak is at 72 h.

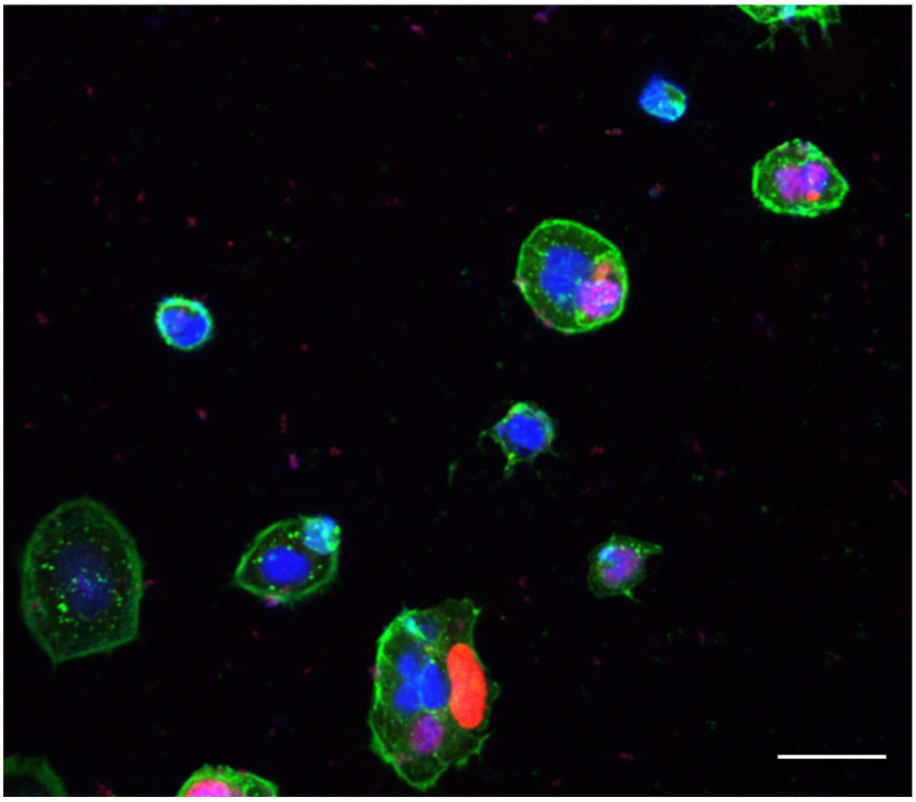

Figure S3. Transfection control immunofluorescence. TAp63 Immunofluorescence in CaCo-2 cells. In the acquisition, are visible transfected and not transfected caco-2 cells stained with the anti p63 antibody (red, ab735 antibody). In green the cytoplasmic localization of TPM4. Scale bar 20 μm.

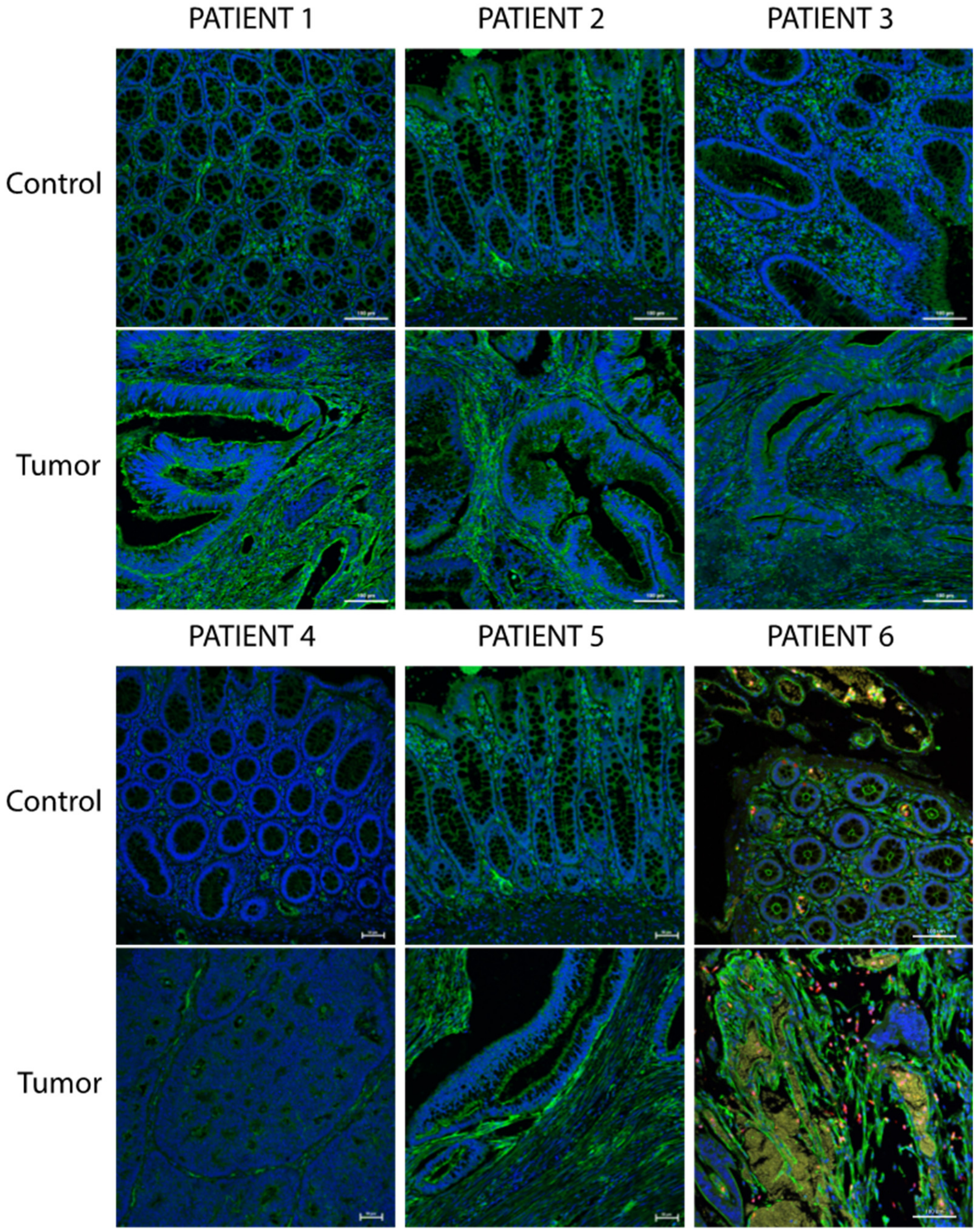

Figure S4. Additional patients immunofluorescence staining. TPM4 (green)/ Dapi (blu). Comparison between tumor adjacent normal area (upper panel) and colon cancer tissue (lower panel) for each patient. Immunofluorescence analysis shows high cytoplasmic levels of TPM4 (green) in neoplastic lesions.The last two acquisitions for patient 6, show an example of DAPI (Blu)/TPM4(green)/KI67 (red) triple staining underlining the high proliferation of colon cancer cells in neoplastic area where TPM4 is strongly detected. Scale Bar: 100 μm (sections of patient 1, 2, 3, 6) and 50 μm (sections of patient 4, 5).
